# Physician’s Perception Versus Patient’s Actual Incidence of Drug Non-adherence in Chronic Illnesses

**DOI:** 10.7759/cureus.1893

**Published:** 2017-11-29

**Authors:** Amber Siddiqui, Anum S Siddiqui, Masood Jawaid, Kamran Ali Zaman

**Affiliations:** 1 Dow University of Health Sciences, Jinnah Sindh Medical University (SMC); 2 Clinical Research Associate, Clinision, Pharmevo Pvt. Limited; 3 Medical Affairs, Pharmevo; 4 Clinical Research Assistant, Pharmevo Pvt. Limited

**Keywords:** medication adherence, mmas, chronic illnesses, polypharmacy, medication cost, morisky medication adherence scale

## Abstract

Objectives

Treatment adherence is crucial to the success of a management plan. The objectives of this study are (i) to assess medication adherence in patients with chronic diseases, (ii) to assess if physicians correctly perceive medication adherence among said patients, and (iii) to investigate the factors associated with low drug adherence.

Materials & methods

This observational cross-sectional study included 283 patients and 208 physicians from various hospitals in Karachi, Pakistan. The participants in the “patient group” completed the eight-item Morisky Medication Adherence Scale form. The participants in the “physician group” completed a questionnaire with questions related to their perception of their patients’ characteristics of adherence to medical prescriptions. Data were entered and analyzed using the Statistical Package for Social Sciences (SPSS) for Windows, Version 22.0. (IBM Corp., Armonk, NY).

Results

The actual incidence of low drug adherence among patients with chronic diseases is 85%. However, the perceived incidence by physicians is 40%. Low adherence was common in women, individuals aged 35 to 50 years, and individuals who were single and illiterate. Adherence decreased with an increasing number of pills, duration of treatment, and increasing average expense of medications. The actual most common barrier to adherence among patients is medication cost; however, physicians perceive forgetfulness to be the more common barrier.

Conclusion

Patients with chronic illnesses have low medication adherence levels. Physicians, however, misinterpret the frequency of low adherence. Our findings will help physicians have a more real and accurate understanding of the challenges their patients face in long-term adherence to treatment regimens. It may ultimately lead to improved treatment adherence and quality of management once patients’ actual challenges are addressed and necessary steps are taken.

## Introduction

The selection of appropriate investigations and treatment regimen forms the basis of disease management in the practice of medicine. However, the role of patient compliance/treatment adherence in the success of a management plan should not be underestimated [1].

The World Health Organization defines compliance as the degree of accuracy to which a patient follows the medical advice of his/her healthcare provider (HCP). Treatment adherence is defined as the degree to which the person’s behavior correlates with the agreed upon recommendations from his/her HCP. Both the terms are interrelated; however, “compliance” suggests the patient is passively abiding by the orders of the physician, while “adherence” highlights how the patient is also a part of the management plan, making it the preferred term [2].

Even when used as interchangeable terms, their implications remain different. Steiner and Earnest [3] have rightly stated these terms “exaggerate the physician’s control over the process of taking medications.” No single term can successfully encompass the wholesome phenomenon of abiding by physician recommendations for chronic illness sufferers.

Meanwhile, some writers have segregated adherence as primary and secondary. Primary non-adherence is the failure of initiation of medical therapy, while secondary non-adherence is the failure of adequately abiding by the medical therapy once initiated [4].

Although treatment adherence is a wider terminology that encompasses both pharmacological and nonpharmacological recommendations of HCPs, for this study, only drug adherence was taken into consideration—a key factor associated with the success of pharmacological therapy. Patients are drug adherent when they take their prescribed medications at given dosages and timings as recommended by their HCPs [5]. Non-adherence to medications is a widely recognized phenomenon and has presented as a major public health concern which significantly contributes to morbidity, mortality, and healthcare costs [6]. Adherence or non-adherence to medications is governed by factors falling into five categories: socioeconomic factors, therapy-related factors, patient-related factors, condition-related factors, and health system/healthcare team-related factors [2].

Identifying medication adherence-related behaviors among patients with chronic illnesses is the foremost step towards obtaining adequate evidence on the consequences, predictors/risk factors, and strategies to improve medication adherence. For this purpose, numerous direct and indirect tools have been proposed. Adherence assessing tools can be subjective (e.g., self-reported by the patient or caregivers) or objective (e.g., counting pills, examining pharmacy refill records, using electronic medication event monitoring systems, or biochemical in which serum or urine drug levels are measured).

An accurate measurement of medication adherence can be quite challenging with its parameters carefully delineated and appropriated for customized situations. The selected tool must be valid, consistent, reliable, and sensitive to change. Hence, there remains no single gold standard tool to measure medication adherence, which itself is a barrier to treatment adherence research [7]. The choice of a tool depends on the individual attributes and goals of the study, the extent of resources available for the study, the clinical setting, the patient’s acceptance, and the convenience of the method. Research suggests medication non-adherence must be taken as a diagnosable and treatable medical condition, and for its diagnosis, a patient-centered approach remains the most accomplished one [8].

Hence, to assess patient adherence to physician-prescribed medication in this study, an adherence-specific self-report questionnaire from the literature, the Morisky Medication Adherence Scale (MMAS), was used [9]. Physicians’ perceptions of drug adherence among their patients were also compared to the patients’ actual drug adherence, which is a lesser studied phenomenon.

## Materials and methods

In this observational cross-sectional study, 283 patients and 208 physicians participated from various public and private hospitals of Karachi, Pakistan after providing informed consent.

The participants included in the “patient group” were ≥ 20 years old and suffered from chronic illnesses, such as diabetes mellitus (DM), hypertension (HTN), ischemic heart disease (IHD) (diagnosed for more than five years), or a chronic physiological condition, such as pregnancy. The participants in the “patient group” completed a questionnaire which included their sociodemographic data, clinical characteristics of their diseases, and the characteristics of their medical prescription. This group also completed the eight-item MMAS, a simple and reliable tool to identify the behavior of patients associated with adherence to prescribed medications.

The MMAS was originally administered in patients with HTN; where the results were reliable (α = 0.83), a significant correlation was seen with blood pressure control (P < 0.05), and the tool proved to be 93% sensitive in detecting patients with poor blood pressure control [10]. Response choices were No/Yes for the first seven items and a five-point Likert response scale for the eighth item. The response “No” was given one point while a “Yes” response was given zero points, except for the fifth item where a “No” response was given a score of zero while a “Yes” response was given a score of one point. For the eighth item, if the participant chose response “A,” the score was one point, and if they chose response “E” the score was zero points. Responses “B, C, and D” were scored “0.25, 0.50, and 0.75”, respectively. The total score on the MMAS-8 can range from 0 to 8, with scores of < 6, 6 to < 8, and 8 reflecting low, medium, and high adherence, respectively.

The participants in the “physician group” completed a questionnaire which included questions related to their perception of their patients’ characteristics of adherence to medical prescription. Data were entered and analyzed using IBM SPSS Statistics for Windows, version 22.0. (IBM Corp., Armonk, NY). MMAS responses were analyzed and compared with patient sociodemographic and clinical characteristics. Physicians’ perceptions of patient medication adherence patterns and barriers to adherence were assessed and compared to the actual pattern of patient medication adherence to deduce if the physicians were accurately aware of the behavior of their patients.

## Results

Of the 283 patients in the study, 37.8% (n = 107) were men and 62.2% (n = 176) were women. Their mean age was 37 years with an age range of 20 to 76 years. Low drug adherence was reported by 84.8% of the sample, 12.4% had medium adherence, and 2.8% showed high drug adherence according to the MMAS assessment. The sociodemographic characteristics of these patients, as compared with their MMAS score, showed that most low drug adherent patients were women, aged 20 to 35 years, married, and attending a university (Table 1).

**Table 1:** Patient Baseline Characteristics and Associated Drug Adherence According to the Morisky Medication Adherence Scale (MMAS)

PATIENT CHARACTERISTICS n = 283 (100%)	LOW ADHERENCE n = 240 (84.8%)	MEDIUM ADHERENCE n = 35 (12.4%)	HIGH ADHERENCE n = 8 (2.8%)
GENDER	n (%)	n (%)	n (%)
Male	89 (83.2%)	14 (13.1%)	4 (3.7%)
Female	151 (85.8%)	21 (11.9%)	4 (2.3%)
AGE			
20-35 years	120 (84.5%)	20 (14.1%)	2 (1.4%)
35-50 years	66 (85.7%)	9 (11.7%)	2 (2.6%)
51-65 years	41 (85.4%)	4 (8.3%)	3 (6.3%)
> 65 years	13 (81.3%)	2 (12.5%)	1 (6.2%)
MARITAL STATUS			
Single	63 (88.7%)	8 (11.3%)	0 (0%)
Married	163 (82.7%)	26 (13.2%)	8 (4.1%)
Divorced	6 (85.7%)	1 (14.3%)	0 (0%)
Widow	8 (100%)	0 (0%)	0 (0%)
EDUCATION			
Nil	7 (87.5%)	1 (12.5%)	0 (0%)
Primary	38 (84.4%)	5 (11.2%)	2 (4.4%)
Secondary	61 (82.4%)	10 (13.5%)	3 (4.1%)
University	134 (85.9%)	19 (12.2%)	3 (1.9%)

The clinical characteristics of these patients, according to their MMAS score showed that 80% to 88% of people with diabetes, hypertension, and pregnant women were low drug adherent, while almost 95% of people with IHD were low drug adherents. Individuals taking more than five medications per day, being treated for two to five years, spending 5,000 Rupees (Rs) to 10,000 Rs monthly on medicines, and those who buy their medicines from private pharmacies were most prone to low drug adherence (Table 2).

**Table 2:** Patient Clinical Characteristics and Associated Drug Adherence According to the Morisky Medication Adherence Scale (MMAS)

PATIENT CHARACTERISTICS n = 283 (100%)	LOW ADHERENCE n = 240 (84.8%)	MEDIUM ADHERENCE n = 35 (12.4%)	HIGH ADHERENCE n = 8 (2.8%)
	n (%)	n (%)	n (%)
ASSOCIATED MEDICAL CONDITIONS			
Diabetes mellitus	39 (88.6%)	5 (11.4%)	0 (0%)
Hypertension	39 (81.3%)	5 (10.4%)	4 (8.3%)
Ischemic heart disease	18 (94.7%)	1 (5.3%)	0 (0%)
Pregnancy	81 (84.4%)	14 (14.6%)	1 (1.0%)
Others	63 (82.9%)	10 (13.2%)	3 (3.9%)
HOSPITALIZED IN THE LAST 6 MONTHS	53 (86.9%)	7 (11.5%)	1 (1.6%)
NUMBER OF MEDICATIONS PER DAY			
1	62 (82.7%)	10 (13.3%)	3 (4.0%)
2-3	123 (86.0%)	17 (11.9%)	3 (2.1%)
4-5	43 (82.7%)	7 (13.5%)	2 (3.8%)
> 5	12 (92.3%)	1 (7.7%)	0 (0%)
DURATION OF TREATMENT IN YEARS			
< 1	112 (83.0%)	20 (14.8%)	3 (2.2%)
1-2	62 (84.9%)	10 (13.7%)	1 (1.4%)
3-5	53 (89.8%)	3 (5.1%)	3 (5.1%)
> 5	12 (80.0%)	2 (13.3%)	1 (6.7%)
MONTHLY MEDICATION EXPENSE IN RUPEES			
< 1000	28 (77.8%)	7 (19.4%)	1 (2.8%)
1,000 – 5,000	140 (83.8%)	21 (12.6%)	6 (3.6%)
5,001 – 10,000	63 (91.3%)	5 (7.3%)	1 (1.4%)
> 10,000	9 (81.8%)	2 (18.2%)	0 (0%)
SOURCE OF MEDICATION			
Supplied from public hospital pharmacy	13 (76.5%)	4 (23.5%)	0 (0%)
Bought from a private pharmacy	168 (88.0%)	21 (11.0%)	2 (1.0%)
Bought by a family member	59 (78.7%)	10 (13.3%)	6 (8.0%)

This study included 208 medical practitioners who practiced general medicine, general surgery, cardiology, and gynecology and obstetrics. When inquired about the drug adherence rate among their patients, they perceived only 39% of their patients were low drug adherent as compared to the actual 85%. Our study observed a substantial difference in physicians’ knowledge of patient drug adherence, patient medication preferences, and patient barriers to adequate drug adherence. A comparison of the views of these two study groups is shown in Table 3.

**Table 3:** Comparison of Physician’s Perception and Patient’s Actual Pattern of Drug Preferences and Adherence

PHYSICIANS' PERCEPTION OF PATIENT’S DRUG PREFERENCES & ADHERENCE n = 208 (100%)	PATIENTS' ACTUAL DRUG PREFERENCES & ADHERENCE n = 283 (100%)
LOW ADHERENCE TO MEDICATIONS	
39.86%	84.8%
AVERAGE DAILY COST (Mean ± SD in Pakistani Rupees)	
345.14 (± 261.15)	3,359.5 (± 2,881.00)
COMMONLY MISSED DRUG DOSE	
Midday (52.9%)	Morning (45.2%)
Morning (31.2%)	Midday (39.6%)
Night (15.9%)	Night (15.2%)
PREFERRED MEDICATION TYPE	
Rapid-acting drugs (88.9%)	Rapid-acting drugs (80.5%)
Delayed action drugs (11.1%)	Delayed action drugs (19.5%)
PREFERRED DAILY MEDICATION DOSE	
Once daily (87%)	Once daily (74.9%)
More than one dose per day (13%)	More than one dose per day (25.1%)
PREFERRED MEDICATION FORM	
Fixed dose combination (77.3%)	Fixed dose combination (58.7%)
Multiple tablets per dose (22.7%)	Multiple tablets per dose (41.3%)

As seen in Table 4, physicians undermined the common barriers decreasing drug adherence among their patients over time. The most common barrier from both physician's and patient's point of view was forgetfulness; however, the youngest age group most commonly forgot to take its medicines. From a patient’s point of view, these barriers were more worrisome, common, and their presence made a patient more prone to low drug adherence (Table 4).

**Table 4:** Comparison of Physician’s Perception and Patient’s Actual Barriers to Drug Adherence

BARRIERS TO DRUG ADHERENCE	PHYSICIAN’S PERCEPTION n = 208 (100%)	PATIENT’S ACTUAL BARRIERS n = 283 (100%)
Forgetfulness	41.8%	59.3%
Alleviated disease severity	22.1%	36.4%
Medication cost	29.5%	84.9%
Medication taste	29.6%	66.2%
Medication side effects	36.8%	83.6%
Medication unavailability	25.6%	25.3%
Complex medication regimen	12.5%	10.9%

## Discussion

This study suggests an 85% incidence of low drug adherence among patients with chronic diseases, which is perceived to be 40% by their physicians. Low adherence was common in women (85.8%), individuals aged 35 years to 50 years (85.7%), individuals who were single (88.7%), and individuals who were illiterate (87.5%). Adherence showed a decreasing trend with an increasing number of pills, duration of treatment, and an increasing average expense of medications. The most common barrier to adherence among patients is medication cost; however, physicians perceived forgetfulness to be the more common barrier, which was not related to increasing age of the population. Even then, physicians undermined the average medication expense of their patients by 10 times.

Our study adopted a dual approach—patient-centered as well as physician-centered. This study is among the few studies that compare patients’ levels of medication adherence to what is perceived by their physicians. It also includes patients with various chronic diseases and chronic physiological conditions (e.g., pregnancy) as compared to many other studies that only assessed medication adherence among patients with HTN [5, 10-12]. However, our study was limited in that we could have involved a larger sample of patients and could have adopted a more precise adherence assessment method, such as counting pills.

Other studies have reported adherence levels at 52% [13] and 65% [14]. However, local data conducted with patients with Type II DM indicate low drug adherence in about 62% cases and were attributed to polypharmacy [15]. Even our study group did not support or prefer polypharmacy. However, another local study among patients with HTN showed a high adherence rate (77%) with improved adherence associated with better awareness and increasing the number of pills prescribed [16]. Nonetheless, our findings are consistent with other studies where the increasing number of pills has shown a decreasing trend in medication adherence [17].

This study compared physicians’ perceptions of patient preferences and barriers to medication adherence, and we found some notable discrepancies. Though other studies report forgetfulness and side effects to be the more common barriers to drug adherence [18-19] (which aligned with our physician group’s perception), the more common adherence barrier in our population was medication expenses. It is impossible for our physicians to correctly estimate the burden of medication expenses on our patients as they highly undermine the cost of their prescribed drugs. The literature also identifies the existence of inconsistent estimation of drug cost by the physicians [20]. Although both our patients and physicians preferred rapid-acting drugs in the form of once-daily dosing and fixed-dose combinations, we could find no substantial studies with which to compare our results.

We recommend more studies with a larger sample and standardized instruments to assess the incongruities among physician perception and patient choices of drug dosages, regimens, and barriers to adherence in order for the physicians to accurately understand the reasons behind such low medication adherence levels. Once the physicians understand these barriers, they can bring about alterations necessary to help make their patients adhere to the medical management of chronic diseases.

## Conclusions

Patients with chronic illnesses have low medication adherence levels. Physicians, however, misinterpret the frequency of low adherence and underestimate the role of medication cost, polypharmacy, and treatment regimen in reduced adherence to medications. These findings should help physicians have a more accurate understanding of the challenges their patients face in long-term adherence to treatment regimens. This understanding may ultimately lead to improved treatment adherence and quality of management once patients’ actual challenges are addressed.
